# Associations between circulating cell-free mitochondrial DNA, inflammatory markers, and cognitive and physical outcomes in community dwelling older adults

**DOI:** 10.1186/s12979-023-00342-y

**Published:** 2023-05-23

**Authors:** Lolita S. Nidadavolu, Danielle Feger, Diefei Chen, Yuqiong Wu, Francine Grodstein, Alden L. Gross, David A. Bennett, Jeremy D. Walston, Esther S. Oh, Peter M. Abadir

**Affiliations:** 1 Division of Geriatric Medicine and Gerontology, Johns Hopkins University School of Medicine, Baltimore, MD USA; 2 Johns Hopkins University Center on Aging and Health, Baltimore, MD USA; 3 Rush Alzheimer’s Disease Center, Rush University Medical Center, Chicago, IL USA; 4 Division of Geriatric Medicine and Gerontology, Johns Hopkins Asthma and Allergy Center, Johns Hopkins University School of Medicine, 5501 Hopkins Bayview Circle, Baltimore, MD 21224 USA

**Keywords:** Cell-free DNA, Inflammation, sTNFR1, Mitochondria, Frailty, Dementia

## Abstract

**Background:**

Dementia and frailty are common age-related syndromes often linked to chronic inflammation. Identifying the biological factors and pathways that contribute to chronic inflammation is crucial for developing new therapeutic targets. Circulating cell-free mitochondrial DNA (ccf-mtDNA) has been proposed as an immune stimulator and potential predictor of mortality in acute illnesses. Dementia and frailty are both associated with mitochondrial dysfunction, impaired cellular energetics, and cell death. The size and abundance of ccf-mtDNA fragments may indicate the mechanism of cell death: long fragments typically result from necrosis, while short fragments arise from apoptosis. We hypothesize that increased levels of necrosis-associated long ccf-mtDNA fragments and inflammatory markers in serum are linked to declines in cognitive and physical function, as well as increased mortality risk.

**Results:**

Our study of 672 community-dwelling older adults revealed that inflammatory markers (C-Reactive Protein, soluble tumor necrosis factor alpha, tumor necrosis factor alpha receptor 1 [sTNFR1], and interleukin-6 [IL-6]) positively correlated with ccf-mtDNA levels in serum. Although cross-sectional analysis revealed no significant associations between short and long ccf-mtDNA fragments, longitudinal analysis demonstrated a connection between higher long ccf-mtDNA fragments (necrosis-associated) and worsening composite gait scores over time. Additionally, increased mortality risk was observed only in individuals with elevated sTNFR1 levels.

**Conclusion:**

In a community dwelling cohort of older adults, there are cross-sectional and longitudinal associations between ccf-mtDNA and sTNFR1 with impaired physical and cognitive function and increased hazard of death. This work suggests a role for long ccf-mtDNA as a blood-based marker predictive of future physical decline.

**Supplementary Information:**

The online version contains supplementary material available at 10.1186/s12979-023-00342-y.

## Background

The rapid advancements in technology and medicine over the past century have led to significant increases in human lifespan. As a result, we have also seen a rise in the occurrence of age-related syndromes, such as Alzheimer’s disease (AD), frailty, and sarcopenia [1]. As people grow older, the likelihood of developing these conditions increases; a 2020 study found that 41% of older adults screened positive for frailty, while 28% screened positive for AD [1].

While increasing age is a major risk factor for AD, frailty, a geriatric syndrome characterized by reduced resilience to physical and psychosocial stressors, also has a significant impact on AD progression [2–4]. A recent study conducted using the UK Biobank data discovered a strong association between frailty and the development of AD; frail individuals, on average, developed AD 3.6 years earlier than their non-frail counterparts [2].

Given the profound connection between age-related cognitive and physical decline, it is crucial to investigate the common biological factors contributing to the progression of geriatric syndromes, including AD and frailty. By doing so, we can better identify those at high risk of developing these conditions and work towards improved prevention and treatment strategies.

A commonly observed feature in both AD and frailty is the chronically low-grade elevation in pro-inflammatory markers such as interleukin-6 (IL-6), interleukin-1β (IL-1β), C-reactive protein (CRP), and tumor necrosis factor alpha (TNF⍺) [5–7]. Numerous studies have examined inflammatory marker elevations in both blood and cerebrospinal fluid of individuals with preclinical AD and a subset of them show promise as an easy to measure biomarker indicating disease risk and progression [8–10]. Elevated plasma soluble tumor necrosis factor alpha receptor 1 (sTNFR1) has shown associations with progression to mild cognitive impairment in cognitively healthy individuals [10]. Despite these associations with inflammation, the biological mechanisms underlying the elevations in these pathways remain poorly understood.

A potential mediator of chronic inflammation in both cognitive and physical decline is circulating cell-free DNA (ccf-DNA), specifically ccf-DNA originating from mitochondrial DNA sources (ccf-mtDNA). Mitochondrial DNA can trigger innate immune system DNA sensors, and its presence acts as damage associated molecular patterns (DAMPs), which are intracellular components released in the setting of tissue and cell injury. In addition to functioning as a DAMP, ccf-mtDNA can mimic some of the characteristics of bacterial and viral nucleic acids. Mitochondrial DNA demonstrates different methylation patterns and hypomethylation of CpG regions relative to genomic DNA, which allow it to be recognized as non-self, further contributing to immune activation [11].

Cell death processes lead to the generation of different ccf-mtDNA sizes. Apoptosis leads to shorter DNA fragmentation patterns (short ccf-mtDNA), while necrosis typically results in variable, less-regulated DNA fragmentation, leading to long ccf-mtDNA fragments [12]. Necrosis, along with other inflammatory cell death processes like pyroptosis, results in the release of cell contents, which generate various immune-stimulating DAMPs [13]. It is plausible that this release of DAMPs could contribute to a persistent low-grade inflammation linked to age-related physical and cognitive decline [14, 15].

In addition to cell death, cell homeostatic mechanisms such as mitophagy are also associated with release of mtDNA into circulation [16]. Mitophagy and the related cell homeostasis process of autophagy have been well studied as a mechanism that is disrupted in aging and neurodegenerative diseases, leading to impaired energy utilization and cellular dysfunction [17]. With a variety of mechanisms resulting in ccf-mtDNA release, prior studies have demonstrated associations between cell necrosis and apoptosis with fragment size. Therefore, quantifying relative sizes of ccf-mtDNA can lead to better understanding of different types of cell turnover processes in aging adults.

Prior studies demonstrate associations between higher levels of plasma ccf-mtDNA with increased mortality and morbidity in several acute diseases [18–20]. Additionally, elevated ccf-mtDNA is associated with poorly controlled chronic diseases that are characterized by cellular stress, including atrial fibrillation, type 2 diabetes and major depressive disorder [21–23]. Older adults with both depression and frailty demonstrate even higher levels of ccf-mtDNA than individuals with either condition alone [24]. Prior studies in cerebrospinal fluid have showed varying trends with respect to levels of ccf-mtDNA in AD [25, 26]. However, additional studies with larger sample sizes are needed to examine associations between cognitive decline and ccf-mtDNA in peripheral blood, and their relationships with previously identified peripheral inflammatory markers (IL-6, CRP, and TNF⍺).

The goal of this study is to identify relationships of serum ccf-mtDNA and inflammatory markers with longitudinal cognitive and physical outcomes in a pooled sample of three community-based cohorts of older adults. We hypothesize that ccf-mtDNA, specifically necrosis-associated long ccf-mtDNA fragments are associated with adverse cognitive and physical outcomes, both cross-sectionally and longitudinally.

## Results

The populations used in this study are three longitudinal clinical-pathologic cohort studies of aging and Alzheimer’s disease based at the Rush Alzheimer’s Disease Center: Religious Orders Study (ROS); Memory and Aging Project (MAP); and Minority Aging Research Study (MARS). Descriptive statistics of the study population are in Table 1. Mean age of participants at baseline was 80 years old and participants were predominantly female, white, and completed post-secondary education. Mean concentrations of short ccf-mtDNA and long ccf-mtDNA were 390 copies/µl and 160 copies/µl, respectively (Table 1). Box plots with the distribution of untransformed ccf-mtDNA values are provided as Supplementary Fig. 1. Mean concentrations of inflammatory markers for individuals at baseline were as follows: CRP = 1.3 (± 0.8) µg/ml; sTNFR1 = 7.4 (± 0.4) pg/ml; TNF⍺ = 1.5 (± 0.6) pg/ml; and IL-6 = 0.9 (± 0.6) pg/ml (Table 1). Percent of individuals with missing data included: global cognition (9.2%); frailty (18%); grip strength (38.5%); gait speed (19.3%); motor gait (14.3%); motor composite score (16.4%). Twenty-seven participants from the starting 672 individuals died over the course of this study.

**Table 1:** Descriptive data of participants at baseline (N = 672) ^a^

	Entire cohort
**Demographics**	
Participants, N (%)	672
Age, mean (SD)	80.4 (7.2)
Sex (Female), N (%)	544 (81.0)
Race (white), N (%)	513 (76.3)
Years of Education, mean (SD)	15.9 (3.5)
**Cognitive testing**	
Global cognitive function (t-score), mean (SD)	50 (10) (N = 607)
Working memory	50 (10) (N = 606)
Semantic memory	50 (10) (N = 600)
Perceptual speed	50 (10) (N = 597)
Perceptual orientation	50 (10) (N = 593)
Episodic memory	50 (10) (N = 605)
**Clinical Diagnosis at ccf-mtDNA Visit**	
No cognitive impairment (NCI)	633 (94.2)
Mild cognitive impairment (MCI)	17 (2.5)
Alzheimer’s Dementia (AD)	18 (2.7)
**Physical measures**	
Frailty z-score, mean (SD) ^b^	-0.07 (0.55) (N = 548)
Grip strength (lbs), mean (SD)	46.1 (16.9) (N = 411)
Gait speed (m/s), mean (SD)	0.64 (0.20) (N = 539)
Motor gait z-score, mean (SD) ^c^	1.02 (0.26) (N = 576)
Motor composite z-score, mean (SD) ^d^	1.00 (0.25) (N = 559)
**CCF-mtDNA**	
Short ccf-mtDNA (1000 copies/µl), mean (SD)	0.39 (0.29)
Long ccf-mtDNA (1000 copies/µl), mean (SD)	0.16 (0.17)
**Inflammatory markers**	
C-Reactive Protein (µg/ml), mean (SD)	1.3 (0.8) (N = 658)
sTNFR1(pg/ml), mean (SD)	7.4 (0.4) (N = 657)
Tumor necrosis factor ⍺ (pg/ml), mean (SD)	1.5 (0.6) (N = 659)
Interleukin-6 (pg/ml), mean (SD)	0.9 (0.6) (N = 659)

Abbreviations: AD = Alzheimer disease; ccf-mtDNA = circulating cell-free mitochondrial DNA; sTNFR1 = soluble Tumor necrosis factor ⍺ receptor 1

Note that ccf-mtDNA variables and inflammatory markers were log-transformed

^a^Missing data as follows: Global cognition: 62 (9.2%); Frailty: 122 (18%); Grip strength: 259 (38.5%); Gait speed: 130 (19.3%); Motor gait: (14.3%); Motor composite: 110 (16.4%)

^b^Frailty is composite variable consisting of grip strength, timed walk, body mass index and fatigue. Larger values correspond to higher frailty

^c^Motor gait is a z-standardized composite variable comprised of walking time, walking steps, turning time, and turning steps. Larger values correspond to less time and fewer steps to complete task

^d^Motor composite score is calculated using the following: Purdue Pegboard Test, Finger-tapping test, Time to cover 8 feet, Number of steps required to cover 8 feet, 360 degree turn time, Number of steps to complete a 360 degree turn, Leg stand, Toe stand, Grip strength, Pinch strength. Larger values correspond to better functionality

### Cross-sectional analyses

Given that ccf-mtDNA can activate the immune system [27], we examined cross-sectional associations between ccf-mtDNA and inflammatory markers in serum. We found significant associations between short ccf-mtDNA fragments and all four inflammatory markers (CRP, β = 0.47 (CI 0.25, 0.69); TNF⍺, β = 0.15 (CI 0.07, 0.23); sTNFR1, β = 0.51 (CI 0.33, 0.70); IL-6, β = 0.41 (CI 0.26, 0.56)), as well as significant associations between long ccf-mtDNA fragments and CRP, TNF⍺, and IL-6 (Table 2). There was no association between long ccf-mtDNA and sTNFR1.

**Table 2:** Associations of ccf-mtDNA with inflammatory markers (N = 672)

Outcomes	Short ccf-mtDNA β (95% CI)	Long ccf-mtDNA β (95% CI)
CRP	**0.47 (0.25, 0.69)**	**0.61 (0.20, 1.02)**
sTNFR1	**0.15 (0.07, 0.23)**	0.14 (-0.02, 0.29)
TNF⍺	**0.51 (0.33, 0.70)**	**0.61 (0.30, 0.92)**
IL-6	**0.41 (0.26, 0.56)**	**0.46 (0.21, 0.70)**

Abbreviations: CI = Confidence interval; CRP = C-Reactive protein; ccf-mtDNA = circulating cell-free mitochondrial DNA; IL-6 = interleukin-6; TNF⍺ = Tumor necrosis factor ⍺; sTNFR1 = soluble Tumor necrosis factor ⍺ receptor 1

Coefficients represent the difference in the outcome per 1000 copies per µL change in the ccf-mtDNA predictor on the log scale. Inflammatory variables were log-transformed. Generalized estimating equations (GEE) were used to estimate the association between ccf-mtDNA and inflammatory markers. Each coefficient was estimated from a separate GEE model. All models adjusted for baseline age, sex, race, and education. Bold indicates p < 0.05

We next identified associations between ccf-mtDNA and cognitive and physical outcomes measured in the ROS-MAP and MARS study, specifically global cognition and scores for cognitive domains as well as physical functioning measures including frailty, grip strength, gait speed and composite motor scores. No significant cross-sectional associations were found between ccf-mtDNA and cognitive and physical function when adjusting for inflammatory markers (Table 3).

**Table 3:** **Associations of ccf-mtDNA with cognitive and physical outcomes adjusting for inflammatory biomarkers (N = 672)**

Outcomes	Short ccf-mtDNA β (95% CI)	Long ccf-mtDNA β (95% CI)
**Cognition**		
Global cognition	-1.34 (-4.51, 1.84)	0.52 (-4.92, 5.96)
Working memory	0.02 (-2.75, 2.79)	1.37 (-3.82, 6.56)
Semantic memory	-1.05 (-4.17, 2.08)	1.41 (-4.00, 6.82)
Perceptual speed	-1.91 (-4.78, 0.95)	1.19 (-3.86, 6.24)
Perceptual orientation	-1.95 (-4.60, 0.70)	-0.64 (-5.65, 4.36)
Episodic memory	-1.02 (-4.39, 2.35)	-0.33 (-5.78, 5.12)
**Physical Function**		
Frailty ^a^	-0.05 (-0.21, 0.11)	-0.21 (-0.48, 0.06)
Grip strength	-2.03 (-7.78, 3.71)	1.36 (-7.19, 9.90)
Gait speed	-0.02 (-0.08, 0.03)	0.02 (-0.07, 0.11)
Motor gait	-0.03 (-0.09, 0.03)	0.06 (-0.06, 0.18)
Motor composite score ^b^	-0.03 (-0.08, 0.03)	0.05 (-0.06, 0.16)

Abbreviations: CI = Confidence interval; ccf-mtDNA = circulating cell-free mitochondrial DNA.

^a^Frailty is composite variable consisting of grip strength, timed walk, body mass index and fatigue. Larger values correspond to higher frailty

^b^Motor gait is a z-standardized composite variable comprised of walking time, walking steps, turning time, and turning steps. Larger values correspond to less time and fewer steps to complete task

^c^Motor composite score is calculated using the following items: Purdue Pegboard Test, Finger-tapping test, Time to cover 8 feet, Number of steps required to cover 8 feet, 360 degree turn time, Number of steps to complete a 360 degree turn, Leg stand, Toe stand, Grip strength, Pinch strength. Larger values correspond to better functionality

Coefficients represent the difference in the outcome per 1000 copies per µL change in the ccf-mtDNA predictor on the log scale. Scores on cognition were t-standardized. Generalized estimating equations (GEE) were used to estimate association between ccf-mtDNA and cognitive and physical outcomes. Each coefficient was estimated from a separate GEE model. All models adjusted for baseline age, sex, race, education, baseline levels of CRP and sTNFR1. Bold indicates p < 0.05

Prior studies have shown associations between frailty and cognition with elevated inflammatory cytokines (IL-6 and sTNFR1) [10, 28]. Therefore, we next examined cross-sectional associations between inflammatory markers and physical and cognitive outcomes in our cohort, and saw associations with lower grip strength, reduced gait speed, and increased frailty scores (Table 4). In particular, soluble TNFR1 demonstrated statistically significant associations with all cognitive and physical measures in our study and CRP also demonstrated significant associations with cognitive and physical measures. Elevated sTNFR1 levels were associated with lower cognition scores in all categories, and worse physical function measures. CRP was significantly associated with semantic memory, perceptual speed and perceptual orientation and the composite measures of motor gait and motor composite score. Elevated levels of IL-6 was only associated with reduced motor composite score.

**Table 4:** Associations of peripheral inflammatory markers with cognitive and physical outcomes (N = 672)

Outcomes	CRP β (CI)	sTNFR1 β (CI)	TNFα β (CI)	IL-6 β (CI)
**Cognition**				
Global cognition	-0.83 (-1.91, 0.25)	**-5.85 (-8.63, -3.08)**	-1.01 (-2.32, 0.31)	-0.87 (-2.36, 0.62)
Working memory	-0.81 (-1.82, 0.20)	**-4.01 (-6.46, -1.56)**	-0.42 (-1.66, 0.82)	-0.67 (-1.94, 0.60)
Semantic memory	**-0.96 (-1.92, -0.01)**	**-5.52 (-7.90, -3.13)**	-0.24 (-1.61, 1.13)	-0.86 (-2.37, 0.64)
Perceptual speed	**-1.26 (-2.34, -0.19)**	**-5.47 (-8.03, -2.92)**	-0.81 (-2.14, 0.51)	-1.19 (-3.24, 0.86)
Perceptual orientation	**-1.18 (-2.04, -0.32)**	**-2.95 (-5.25, -0.65)**	**-1.10 (-2.12, -0.08)**	-0.57 (-1.65, 0.51)
Episodic memory	-0.50 (-1.59, 0.59)	**-4.93 (-7.92, -1.94)**	-0.91 (-2.27, 0.44)	-0.89 (-2.17, 0.39)
**Physical Function**				
Frailty ^a^	0.003 (-0.05, 0.05)	**0.15 (0.03, 0.27)**	-0.03 (-0.08, 0.02)	-0.01 (-0.07, 0.05)
Grip strength	**-2.90 (-4.94, -0.86)**	**-9.69 (-15.86, -3.52)**	-0.60 (-2.77, 1.57)	-2.18 (-4.66, 0.30)
Gait speed	-0.01 (-0.03, 0.003)	**-0.08 (-0.13, -0.03)**	-0.01 (-0.04, 0.01)	-0.01 (-0.03, 0.02)
Motor gait ^b^	**-0.02 (-0.05, -0.002)**	**-0.16 (-0.22, -0.10)**	-0.01 (-0.05, 0.02)	-0.02 (-0.05, 0.01)
Motor composite score ^c^	**-0.03 (-0.05, -0.01)**	**-0.18 (-0.23, -0.13)**	-0.02 (-0.04, 0.01)	**-0.03 (-0.06, -0.01)**

Abbreviations: CI = Confidence interval; CRP = C-Reactive protein; ccf-mtDNA = circulating cell-free mitochondrial DNA; IL-6 = interleukin-6; TNF⍺ = Tumor necrosis factor ⍺; sTNFR1 = soluble Tumor necrosis factor ⍺ receptor 1

^a^Frailty is composite variable consisting of grip strength, timed walk, body mass index and fatigue. Larger values correspond to higher frailty

^b^Motor gait is a z-standardized composite variable comprised of walking time, walking steps, turning time, and turning steps. Larger values correspond to less time and fewer steps to complete task

^c^Motor composite score is calculated using the following items: Purdue Pegboard Test, Finger-tapping test, Time to cover 8 feet, Number of steps required to cover 8 feet, 360 degree turn time, Number of steps to complete a 360 degree turn, Leg stand, Toe stand, Grip strength, Pinch strength. Larger values correspond to better functionality

Coefficients represent the difference in the cognitive and physical outcomes per one unit increase in the inflammatory marker on the log scale. Inflammatory variables were log-transformed. Generalized estimating equations (GEE) were used to estimate association between inflammatory markers and cognitive and physical outcomes. Each coefficient was estimated from a separate GEE model. All models were adjusted for baseline age, sex, race, and education. Bold indicates p < 0.05

### Longitudinal analyses

Longitudinal associations between ccf-mtDNA fragments, frailty and global cognitive scores were next examined using random effects modeling over an eight year follow up period. Higher levels of long ccf-mtDNA were associated with steeper declines (worsening) in the motor gait score (walking speed and turning) slopes over time (Table 5).

**Table 5:** Adjusted longitudinal changes in global cognition score and physical function relative to baseline ccf-mtDNA levels (N = 672)

Outcome	Intercept on ccf-mtDNA (95% CI)	Slope on ccf-mtDNA (95% CI)
**Short ccf-mtDNA**		
Global cognition	-1.77 (-4.56, 1.02)	-0.01 (-0.57, 0.56)
Frailty ^a^	0.04 (-0.12, 0.19)	0.02 (-0.001, 0.05)
Gait speed	-0.04 (-0.10, 0.01)	-0.01 (-0.02, 0.004)
Grip strength	-0.29 (-4.40, 3.82)	-0.04 (-0.68, 0.61)
Motor gait ^b^	-0.06 (-0.13, 0.01)	-0.01 (-0.02, 0.002)
Motor composite score ^c^	-0.06 (-0.12, 0.01)	-0.002 (-0.01, 0.01)
**Long ccf-mtDNA**		
Global cognition	3.55 (-0.82, 7.91)	-0.52 (-1.39, 0.34)
Frailty	-0.13 (-0.37, 0.11)	0.03 (-0.01, 0.07)
Gait speed	-0.02 (-0.11, 0.07)	-0.01 (-0.02, 0.01)
Grip strength	2.89 (-3.39, 9.17)	-0.45 (-1.42, 0.52)
Motor gait	-0.01 (-0.11, 0.10)	**-0.02 (-0.03, -0.001)**
Motor composite score	0.002 (-0.09, 0.10)	-0.01 (-0.02, 0.004)

Abbreviations: CI = Confidence interval; ccf-mtDNA = circulating cell-free mitochondrial DNA.

^a^Frailty is composite variable consisting of grip strength, timed walk, body mass index and fatigue. Larger values correspond to higher frailty

^b^Motor gait is a z-standardized composite variable comprised of walking time, walking steps, turning time, and turning steps. Larger values correspond to less time and fewer steps to complete task

^c^Motor composite score is calculated using the following items: Purdue Pegboard Test, Finger-tapping test, Time to cover 8 feet, Number of steps required to cover 8 feet, 360 degree turn time, Number of steps to complete a 360 degree turn, Leg stand, Toe stand, Grip strength, Pinch strength. Larger values correspond to better functionality

Model-estimated intercept and slope parameters from latent growth models of each outcome domain regressed on baseline ccf-mtDNA levels over a mean 8-year period

Intercept on ccf-mtDNA represents the difference in the mean baseline levels of outcome per 1000 copies per uL change in the ccf-mtDNA predictor on the log scale

Slope on ccf-mtDNA represents the difference in the mean rate of change in levels of outcome per 1000 copies per uL change in the ccf-mtDNA predictor on the log scale

Ccf-mtDNA variables are log-transformed. Each row of coefficients was estimated from a separate latent growth model. All models were adjusted for baseline age, sex, race, education, baseline levels of CRP and TNFR1. Bold indicates p < 0.05

Given the strong cross-sectional associations between inflammatory markers and declining physical function, we next examined associations between ccf-mtDNA and inflammatory markers with mortality in this cohort. When adjusting for demographics, higher levels of short ccf-mtDNA was associated with elevated hazard of death (HR = 3.54, CI 1.02–12.30), although this significance did not remain when adjusting for inflammatory variables (Table 6, Model 2). In survival analyses with 4,921.5 person-years, elevated levels of sTNFR1 were associated with a higher hazard of death over an 8-year follow up period when adjusting for demographics (HR = 4.37, CI 1.19–16.00). Long ccf-mtDNA did not have associations with mortality (Table 6, Model 3).

**Table 6:** Associations of ccf-mtDNA and peripheral inflammatory markers with mortality (N = 672)

	Individual model sets Adjusted Hazard Ratio of Death β (95% CI)	Model 2 Adjusted Hazard Ratio of Death β (95% CI)	Model 3 Adjusted Hazard Ratio of Death β (95% CI)
Short ccf-mtDNA	**3.54 (1.02, 12.30)**	2.21 (0.55, 8.90)	N/A
Long ccf-mtDNA	2.40 (0.19, 30.7)	N/A	1.93 (0.15, 24.49)
CRP	1.38 (0.84, 2.27)	1.08 (0.63, 1.84)	1.12 (0.66, 1.90)
sTNFR1	**4.37 (1.19, 16.00)**	3.86 (0.96, 15.54)	3.92 (0.95, 16.21)

Abbreviations: CI = Confidence interval; CRP = C-Reactive protein; ccf-mtDNA = circulating cell-free mitochondrial DNA; sTNFR1 = soluble Tumor necrosis factor ⍺ receptor 1

Coefficients represent that for each 1-unit increase in the predictor variable, the hazard of death changes by β times. Each row of coefficients in the Individual model set was estimated from a separate model and was adjusted for baseline age, sex, race, and education. Models 2 and 3 were adjusted for demographics, CRP, and sTNFR1, with inclusion of short ccf-mtDNA (Model 2) and long ccf-mtDNA (Model 3). Ccf-mtDNA variables and peripheral inflammatory variables were log-transformed and modeled as continuous variables. Cox proportional-hazards model was used to estimate the hazard ratios. Bold indicates p < 0.05

## Discussion

This study shows cross-sectional and longitudinal relationships between peripheral inflammatory markers (ccf-mtDNA and sTNFR1), and cognitive and physical decline in a community dwelling population of older adults. Interestingly, short and long ccf-mtDNA were positively associated with most inflammatory markers measured at the same time point, with the exception of no significant association between long ccf-mtDNA and sTNFR1. Additionally, there were no associations between ccf-mtDNA fragments and cognitive and physical outcomes on cross-sectional analysis, but high levels of long ccf-mtDNA fragments were associated with worsening motor gait scores over an eight-year period. Short ccf-mtDNA fragments and sTNFR1were associated with higher hazard of death over an eight year follow up period, but this association went away when adjusting for inflammatory markers.

Compared to prior analysis of genomic-derived ccf-DNA in the ROS-MAP cohort [29], we did not observe cross-sectional associations between ccf-mtDNA fragments and cognitive and physical outcomes. However, longitudinal analysis demonstrated associations with a composite gait measure for the long ccf-mtDNA fragment after adjusting for inflammatory markers (Table 5), suggesting that mitochondrial derived circulating cell-free DNA may be more strongly linked to physical decline, while genomic derived ccf-DNA is linked to both cognitive and physical decline.

We believe that several factors could be contributing to these findings. Firstly, ccf-mtDNA may be degrading too quickly to be accurately detected in our banked serum samples. Previous studies have discussed degradation of circulating longer fragment ccf-mtDNA as a factor contributing to higher levels of detectable smaller ccf-mtDNA fragments in circulation [12, 30].

Secondly, we may find more associations with ccf-mtDNA and physical and cognitive decline when looking at either a younger population or a population with a higher percentage of males. Our study population, though well-characterized, is primarily female, and the mean age is 80. By examining middle-aged and older adults in a more balanced gender distribution, more connections may be uncovered between age-related decline and long ccf-mtDNA levels, both cross-sectionally and longitudinally.

We tested whether mean levels of ccf-mtDNA differ by whether cognitive and physical outcomes are missing using t-tests. We detected no differences for global cognitive function, working memory, semantic memory, perceptual speed, perceptual orientation, episodic memory, and grip strength. Interestingly, we observed in our study population that individuals missing frailty measurements had higher levels of ccf-mtDNA at that visit. The frailty score through the ROS-MAP and MARS studies is a composite of 5 individual tests, two of which require the participant to perform physical tests at the study site. We believe these associations between high ccf-mtDNA and missing frailty scores could be due to individuals with significant functional limitation who were not able to complete the physical tests at the visit. This is also a likely factor contributing to the higher percentage of missing data for gait speed, frailty, and grip strength when compared to the percent missing data for global cognition.

A distinguishing characteristic of our study compared to previous work examining associations between ccf-mtDNA and mortality is that the ROS-MAP and MARS populations includes community dwelling older adults, while other studies were in hospitalized, acutely ill populations [20, 31]. Despite this major difference in the participants sampled and different methods of ccf-mtDNA isolation and measurement, we observed similar associations between elevations in ccf-mtDNA and increased mortality in our study. Increases in circulating DAMPs (ccf-mtDNA) may be contributing to activation of inflammatory pathways which subsequently leads to increased mortality.

An additional consideration with our study is the potential for cellular mitochondrial DNA contamination that could be a result of varying sample processing over the course of the ROS-MAP study. To address these concerns, future studies can use specialized blood collection tubes for cell-free DNA analysis, minimizing this effect.

Soluble TNFR1 has been identified as an inflammatory marker predictive of frailty and increased mortality in other studies [28], and our study reaffirms these findings as well as identifies associations between elevated sTNFR1 and lower cognitive test scores (both global cognitive score and cognitive subdomain scores) [10, 32]. Interestingly, our cross-sectional analysis of inflammatory markers and cognitive testing showed no associations between IL-6 and these cognitive domains, in contrast to other studies [10, 33], and this could be due in part to demographic differences in the study populations. The other studies focused on younger adults (mean age 60) compared to this study (mean age 80), while others had more even distributions of men/women compared to this study (81% female), and others had higher percentages of smokers. Additionally, differences in ELISA assays for quantifying the inflammatory markers could have contributed to the differences in mean cytokine values at baseline in these other studies compared to ours.

A question that remains is whether increases in ccf-mtDNA precede the elevations in inflammatory markers observed in individuals with frailty and cognitive decline, or if the cell death releasing ccf-mtDNA in circulation occurs because of increased inflammation. Follow up studies will also examine the associations between ccf-mtDNA levels with AD biomarkers including amyloid-beta and tau proteins. These questions can be further studied in human cell lines and mouse models of AD.

Studies using human serum at this time are not able to convey information about cellular processes such as mitophagy, that can also contribute to ccf-mtDNA levels. Nonetheless, differentiating between mitophagy and necrosis/apoptosis is crucial for a deeper comprehension of how these processes alter with aging and contribute to the generation of ccf-mtDNA. Additionally, aging studies using animal models with targeted genetic deletions in mitophagy genes can also be used to understand the degree to which mitophagy and autophagy contributes to ccf-mtDNA versus apoptosis or necrosis.

Ccf-mtDNA are released passively and actively into circulation from a wide variety of cell death processes and are known to be immunostimulatory by activating the innate immune system through DNA sensing pathways [11]. Little is known about aging-related changes in these immune pathways, particularly changes in the expression of DNA sensors in peripheral immune cells and the brain of individuals with elevated risk of dementia, which can possibly lead to increased inflammatory activation. Future studies can focus on elucidating this relationship and the role of immunostimulatory ccf-mtDNA in contributing to chronic inflammation. Ultimately, better characterization of the downstream effects of ccf-mtDNA and sTNFR1 on chronic inflammation in AD and frailty can help identify novel drug targets that can reduce the high mortality and morbidity of these syndromes.

## Conclusions

This study identifies serum ccf-mtDNA as a promising biomarker linked to chronic inflammation, which could serve as an indicator for individuals at a heightened risk of physical decline and mortality. Furthermore, we underscore the importance of sTNFR1, which demonstrates a strong connection with cognitive and physical deterioration, an increased likelihood of death, and a relationship with apoptosis-associated short ccf-mtDNA fragment. Future studies will include mechanistic studies to better understand the relationships between ccf-mtDNA fragments and chronic inflammation, particularly how these elements interact with pathways known to be disrupted cognitive and physical decline, thereby enhancing our comprehension of these intricate relationships.

## Methods

### Study sample

Data were obtained from the Rush Alzheimer’s Disease Center Religious Orders Study, Rush Memory and Aging Project (ROS-MAP) and Minority Aging Research Study (MARS), which have been detailed in prior publications [34, 35]. ROS began in 1994 and consists of nuns, priests, and brothers from across the USA, MAP began in 1997 and consists of community-dwelling older adults from the greater Chicago metropolitan area, and MARS began in 2004 and consists of community-dwelling older African Americans free of dementia. All studies were approved by the Institutional Review Board at Rush University Medical Center. All participants signed an informed consent, Anatomic Gift Act, and a repository consent to allow their resources to be shared. Annual assessments are conducted for all participants. Serum samples were obtained from ROS-MAP and MARS participants during cohort evaluations; we included here 672 samples which were available for analysis at the time of this study.

### Biological measures

Digital PCR: The digital PCR method was used to directly quantify mitochondrial-derived ccf-DNA. Two sets of primers were designed targeting a conserved region of the mitochondrial ribosomal 16 S gene (Integrated DNA Technologies, Iowa City, IA); the first primer pair amplified a 79-bp fragment (short ccf-mtDNA, apoptotic fragment) and the second primer pair amplified an adjacent 230-bp fragment (long ccf-mtDNA, necrotic fragment).

Run preparation: Serum was diluted with PBS and heat denatured, then vortexed until the solid pellet broke apart, approximately 3 s. The samples were then spun down in a microcentrifuge for 15 s to pellet any viscous material. 10 µl of the supernatant was mixed with 10 µl of water, vortexed and spun down. One Master mix contained two assays: “long fragment” labeled with Cy5, and “short fragment” labeled with Hex. 16.4 µl of Master mix was mixed with 4 µl of the prepared serum and the entire volume was loaded into the digital PCR plate and run on the Constellation digital PCR system (Formulatrix, Bedford, MA) using the following thermocycling conditions: [98 C for 120 s, 40 cycles of {98 C for 10 s, 60 C for 30 s}, 45 C for 30 s].

Data calling: Amplification thresholds were applied using the Constellation software (Formulatrix, Bedford MA). Results were exported and scripts developed by Formulatrix were run to distinguish between partitions which had amplification with both mitochondrial assays and those which amplified only one.

ELISA: Peripheral inflammatory markers C-reactive protein, sTNFR1, TNF⍺, and IL-6 were assayed in serum from the RADC biorepository. All assays were performed on the Meso Scale Discovery (MSD) V-plex electrochemiluminescence platform at the Research Laboratory Core of the Institute for Clinical and Translational Research at Johns Hopkins University. Interassay coefficients of variation using controls on every plate were as follows: CRP = 8.46%; sTNFR1 = 7.44%; TNF⍺/IL-6 = 5.12%. Inflammatory markers were log transformed and generalized estimating equation (GEE) models were used to determine the relationship between cytokines and cognition and physical function outcomes.

### Cognitive measures

Twenty-one cognitive tests were conducted at each study visit, nineteen of which were used to assess the following cognitive domains: episodic memory, semantic memory, working memory, perceptual orientation, and perceptual speed. Each test had a z-score constructed using all participants and each cognitive domain z-score was calculated by averaging all z-scores within that domain. A global cognitive function t-score was calculated by averaging the z-score of each of the 19 cognitive tests [36].

A three-stage process consisting of computer scoring of cognitive tests, clinical judgement by a neuropsychologist and diagnostic classification by a clinician was used to assess dementia status at each study visit [37, 38]. Additional details on diagnostic criteria for this study is detailed in Nidadavolu et al. [29].

### Physical measures

Physical measures (grip strength, gait speed, body composition, and fatigue, global motor function composite score) were obtained at each study visit to create a frailty composite z-score. Methodologic details are in Nidadavolu et al. [29].

### Adjustment variables

Race in the ROS-MAP and MARS studies was self-reported as White, Black or African American, American Indian or Alaska Native, Native Hawaiian or Other Pacific Islander, Asian, Other, or Unknown. A binary race variable was defined as 0 = White and 1 = any other race; this binary race classification was used in all analyses since there were few participants who reported race other than White or African American. Analyses were adjusted for age, sex, race, and years of education.

### Analyses

We only used complete cases for both predictor and outcome variables in this study. We described the sample initially with descriptive statistics. Generalized estimating equations (GEE) were used to estimate the cross-sectional associations between the log-transformed numbers of copies of short and long ccf-mtDNA fragments with inflammatory markers (i.e. CRP, sTNFR1, TNF⍺, IL-6). Because of the highly skewed nature of inflammatory markers, we log transformed values of all the above-mentioned markers. Similarly, GEE models were used to estimate cross-sectional associations between ccf-mtDNA predictors (i.e. log-transformed numbers of copies of short and long ccf-mtDNA fragments) with cognitive measures (i.e. global cognition, episodic memory, semantic memory, working memory, perceptual speed, and perceptual orientation), as well as frailty and other physical functioning outcomes (grip strength, gait speed, motor composite score). Another set of Generalized estimating equations (GEE) was used to estimate the association between inflammatory markers and cognitive and physical outcomes. Longitudinal associations between ccf-mtDNA and changes in cognitive (global, episodic memory, semantic memory, working memory, perceptual speed, and perceptual orientation) and physical outcomes (frailty, gait speed, grip strength, hand strength, and motor composite score) were evaluated using linear latent growth model, which is analogous to mixed effects models with random effects for participants and time [39]. We then ran separate Cox proportional-hazards models to estimate hazard ratios for mortality, with each ccf-mtDNA, cytokines and physical and cognitive measures as the main predictor. All models were adjusted for baseline age, sex, race, and education. Stata version 17.0 was used for analysis. This study was approved by the Johns Hopkins University and Rush University Medical Center Institutional Review Boards (IRB).

## Electronic supplementary material

Below is the link to the electronic supplementary material.

Supplementary Fig. 1: Boxplots of distributions for the ccf-mtDNA values (number of fragment copies per µl)

## Data Availability

Data are available upon reasonable request to the corresponding authors.

## Abbreviations

AD: Alzheimer’s disease
ccf-mtDNA: Circulating cell-free mitochondrial DNA
CI: Confidence Interval
CRP: C-reactive protein
DAMP: Damage associated molecular patterns
ELISA: Enzyme-linked immunoassay
GEE: Generalized estimating equations
IL-6: Interleukin-6
MAP: Memory and Aging Project
MARS: Minority Aging Research Study
ROS: Religious Orders Study
sTNFR1: Soluble tumor necrosis factor ⍺ receptor 1
TNF⍺: Tumor necrosis factor ⍺
