# Supplementary material for: Associations between circulating cell-free mitochondrial DNA, inflammatory markers, and cognitive and physical outcomes in community dwelling older adults
Source: Immun Ageing. 2023 May 23;20:24. doi: 10.1186/s12979-023-00342-y (PMC10204157; doi:10.1186/s12979-023-00342-y)

Supplementary Figure 1. Boxplots of distributions for the ccf-mtDNA values (number of fragment copies per µl)


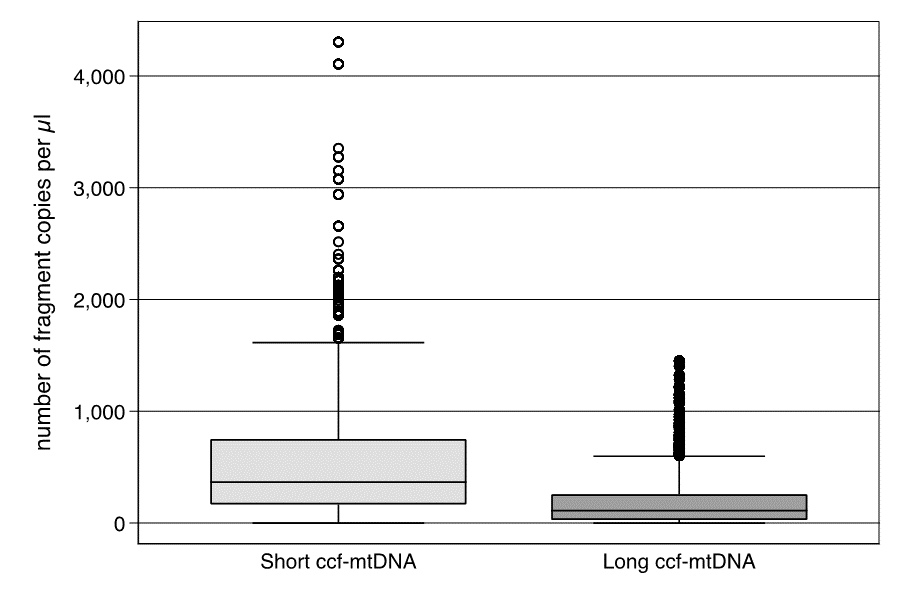

Supplement: Supplementary file 1 — Supplementary Fig. 1: Boxplots of distributions for the ccf-mtDNA values (number of fragment copies per µl) [file 12979_2023_342_MOESM1_ESM.docx]
